# Supplementary material for: Large polarization-dependent exciton optical Stark effect in lead iodide perovskites
Source: Nat Commun. 2016 Aug 31;7:12613. doi: 10.1038/ncomms12613 (PMC5013647; doi:10.1038/ncomms12613)
Supplement: Supplementary Information — Supplementary Figures 1-9, Supplementary Tables 1-2, Supplementary Note 1 and Supplementary References [file ncomms12613-s1.pdf]

Supplementary Figures.

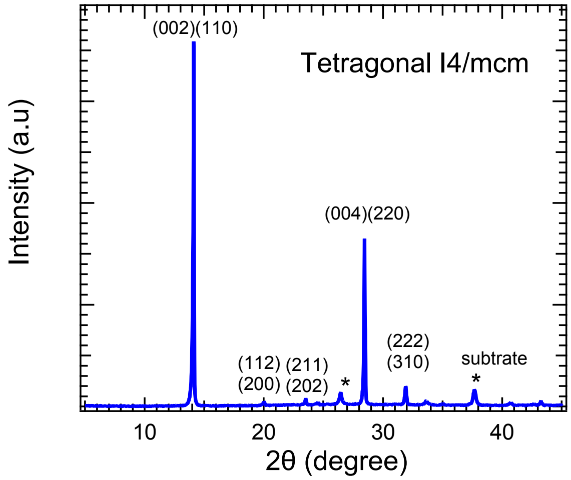

Supplementary Figure 1. X-ray diffraction (XRD) pattern of the sample. The XRD characterization indicates that the lead iodide perovskite sample used in the current study is in tetragonal phase.

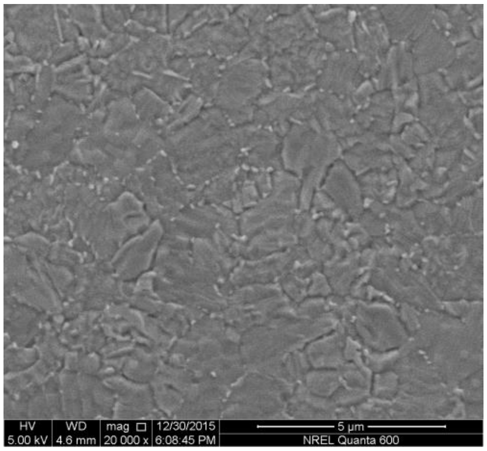

Supplementary Figure 2. Scanning electron microscopic (SEM) Characterization. SEM image of the top-view of the lead iodide perovskite sample used in the current study.

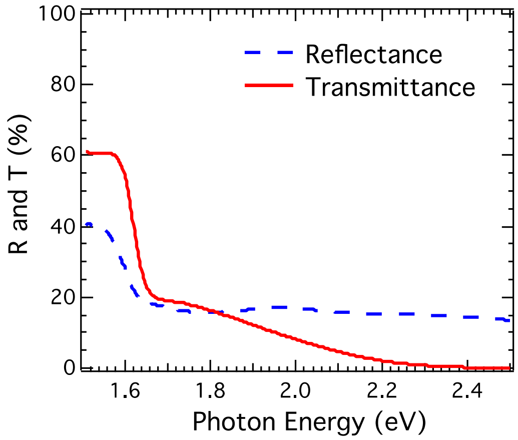

Supplementary Figure 3. Optical reflectance and transmittance of the lead iodide perovskite film. The reflectance (R) and transmittance (T) spectra are collected by a visible-near-infrared spectrometer (UV-3600, Shimadzu). Because the perovskite film is deposited on a transparent quartz substrate in 1 mm thickness, the absorption spectrum (A) of the film can be approximately calculate from A=-log[T/(1-R)].

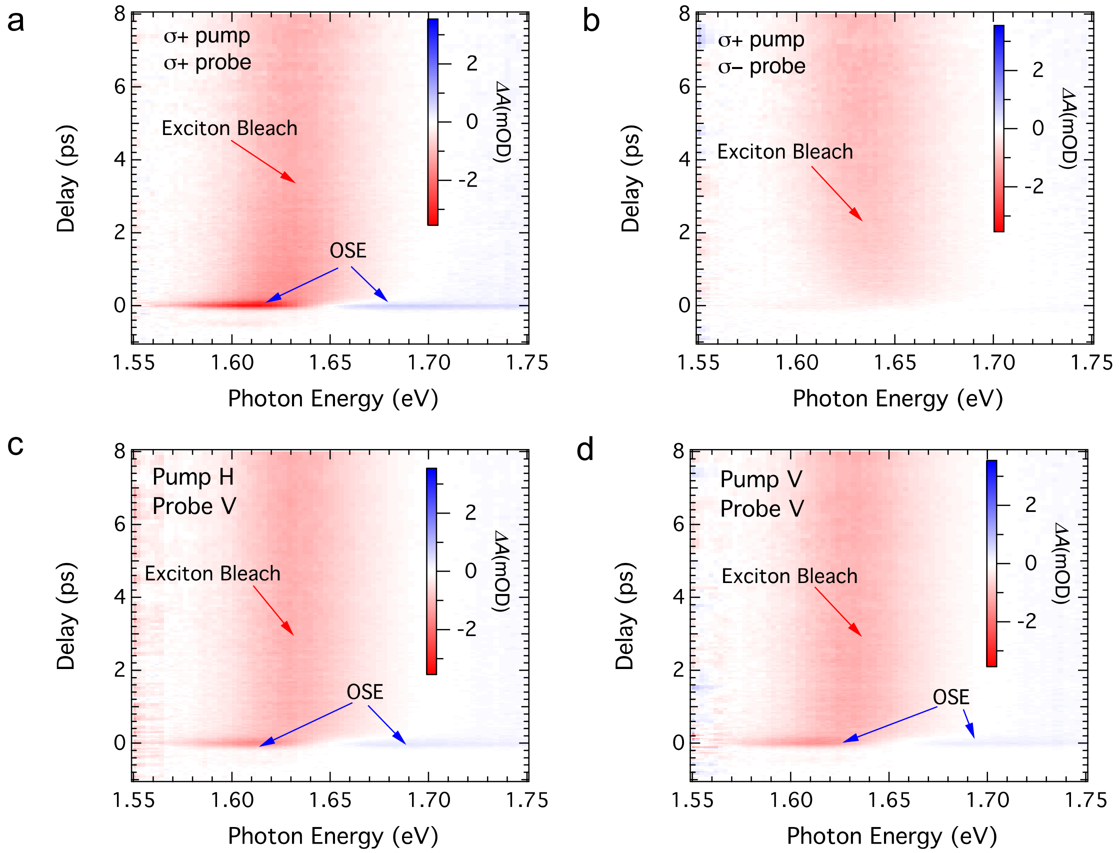

Supplementary Figure 4. Transient absorption (TA) spectra measured under different pump-probe polarization configurations. The pump photon energy is

1.55 eV and is below the exciton resonance energy (1.631 eV). Panel (a) and (b) are the TA spectra measured by the co- (σ+, σ+) and counter-circularly (σ+, σ-) polarized pump-probe configurations, respectively. Panel (c) and (d) are the TA spectra measured using linearly polarized pump-probe beams with perpendicular (HV) and parallel (VV) polarization configuration. Because of the spectral overlap between exciton transition and pump bandwidth, both real and virtue excitations occur. As indicated by the arrows, the derivative-like features at time zero are attributed to the optical Stark effect (OSE) induced absorption change, which results from virtual excitation of the pump. On the other hand, the bleach at longer delay is assigned to the bleaching of the exciton transition caused by the phase-space filling of the charge carriers.

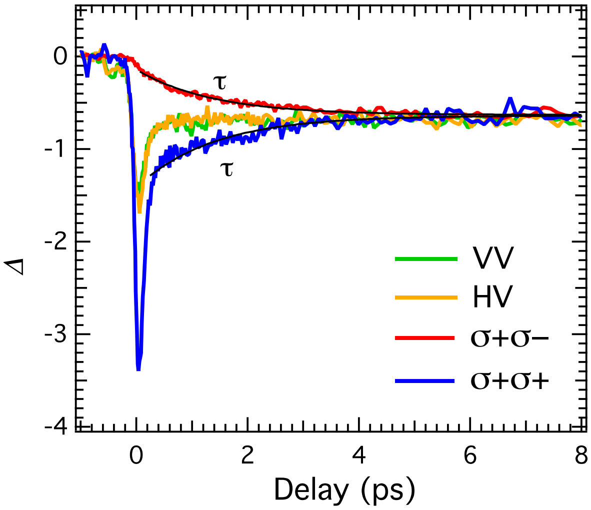

Supplementary Figure 5. Comparison of TA kinetics recorded at 1.61 eV from TA spectra collected from the indicated pump-probe polarization configurations. The kinetic traces for (σ+, σ-) and linearly polarized pump-probe configurations show a sharp spike near time zero, which however is not observed in the kinetics for (σ+, σ-). This spike is attributed to the OSE induced absorption change, and the width of this spike is determined by the correlation time of pump-probe (around 180 fs). For (σ+, σ-), the spike is absent because the OSE induced absorption change cannot be probed due to the selection rule (discussed in text). In addition to the OSE, all the kinetic traces contain residual signals that are caused by the presence of free carriers. A slow formation and decay component are shown before 6 ps in (σ+, σ-) and (σ+, σ+) kinetics, respectively, which have been attributed to exciton spin orientation flip process.[1] Because of the selection rule, only excitons with m_j=-1 (+1) are initially generated by the circularly polarized pump, and a portion of these excitons will convert to the excitons with m_j=+1 (-1) through j-spin flip. Both kinetic traces reach plateau after 6 ps, indicating that the two exciton states are equally populated. The formation and decay time constants are determined to be 1.36 and 1.38 ps, respectively, from single exponential fitting, suggesting the similar spin flip rate for different initial exciton states. For the linear polarized pump-probe, the exciton states with m_j=+1 and -1 are equally populated,

and no net j-spin flip process is observed. The real excitation induced component is constant within 8 ps due to the long lifetime of the carriers. It should be noted that the real excitation induced signals in the equilibrium stage are the same for different pump-probe configurations because the same pump intensity results in the same carrier density. As both spectral shape and kinetics of the TA components due to real carriers are known, the OSE induced TA spectra can then be isolated by subtracting the TA spectra caused by real carriers from the total TA spectra.

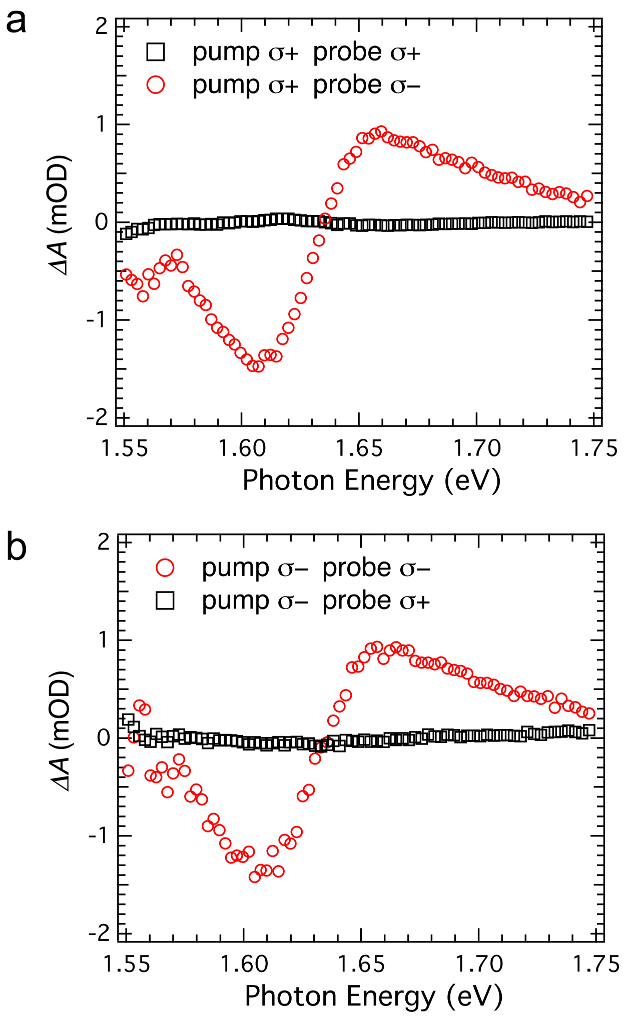

Supplementary Figure 6. Polarization dependent OSE induced TA spectra. (a) OSE induced TA spectra measured by pump-probe configurations of (σ+, σ+) and (σ+, σ-). (b) OSE induced TA spectra measured by pump-probe configurations of (σ-, σ-) and (σ-, σ+).

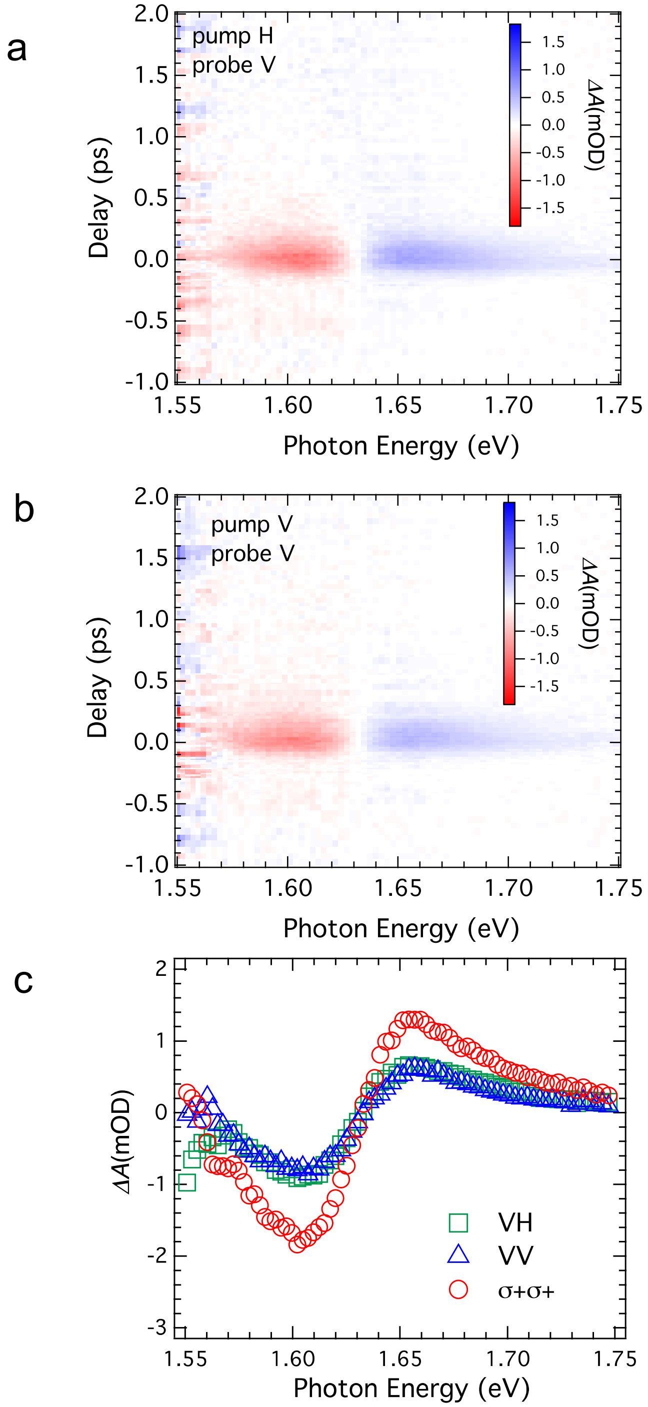

Supplementary Figure 7. OSE induced TA spectra measured by linearly polarized pump probe configuration. Panel (a) and (b) are the OSE induced TA spectra of perovskite films obtained by subtracting the carrier induced bleach from the total TA spectra shown in panel (c) and (d) of supplemental Fig. S3, respectively. Panel (c) is the comparison of the OSE under different pump-probe polarization configurations. The signal magnitude for co-circularly polarized pump-probe configuration is twice as large as that for the linearly polarized pump-probe.

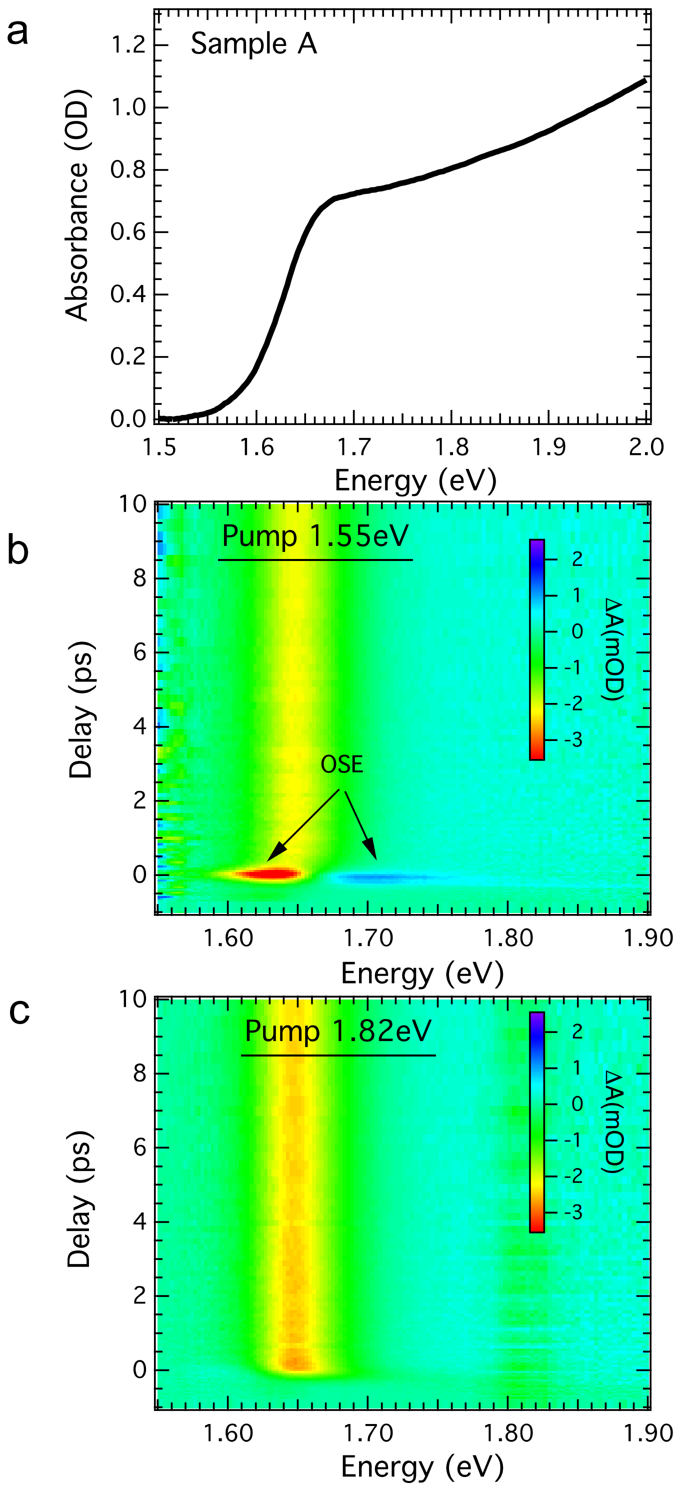

Supplementary Figure 8. Optical Stark effect in the perovskites made from a different method (sample A). Sample A was prepared differently from the sample measured in the main text. The synthesis method of sample A was described in a reported literature.[2] (a) The absorption spectrum of sample A. (b) For the below

bandgap excitation, OSE caused spectral features are clearly shown around time zero, (c) while the OSE features are absent for the above bandgap excitation.

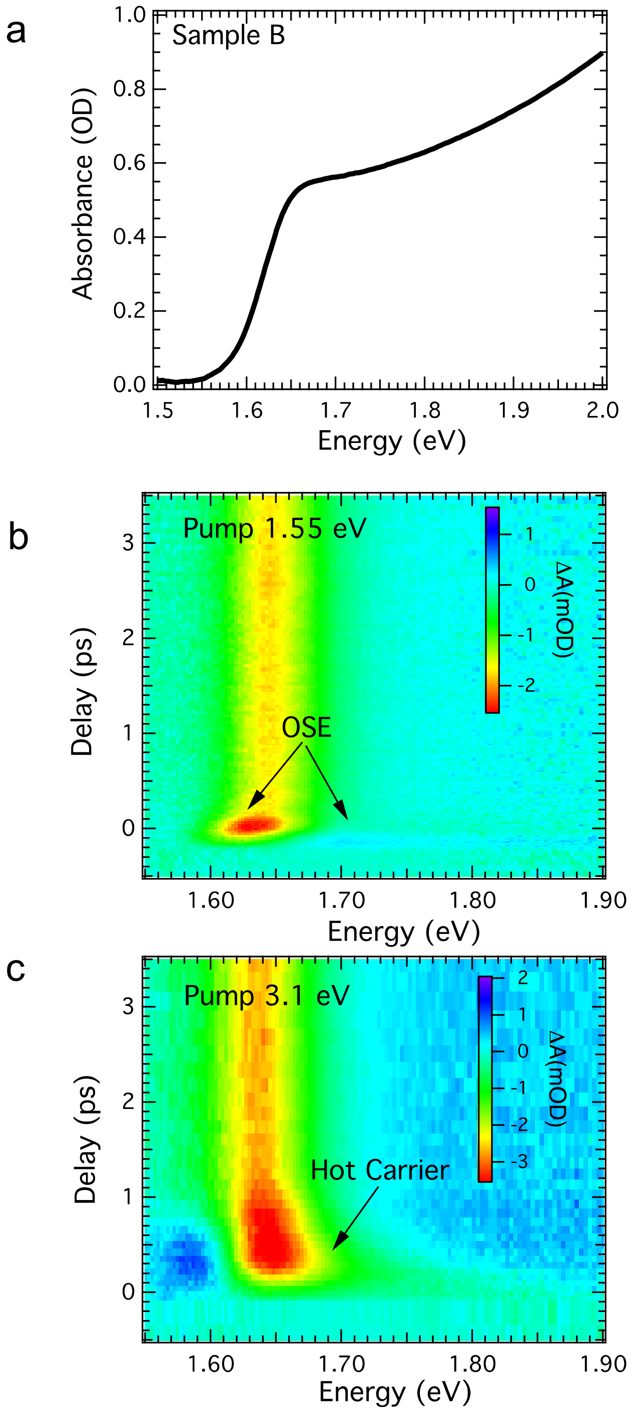

Supplementary Figure 9. Optical Stark effect in the perovskites made from a different method (sample B). Sample B was prepared differently from sample A or the sample measured in the main text. The synthesis method of sample A was described in another reported literature.[3] (a) The absorption spectrum of sample A.

(b) For the below bandgap excitation, OSE caused spectral features are clearly shown around time zero, (c) while the OSE features are absent for the above bandgap excitation. In contrast of a sharp bleach band at lower energy side of bandgap at time zero, the TA shows a broad bleach band at higher energy side of the bandgap at short delay, which is attributed to the hot carrier band-filling effect due to the high excitation energy.[2]

Supplementary Tables

Supplementary table 1. List of Parameters for the absorption coefficient fitting shown in Fig. 2 in the main text.

E_g (eV)	R_{ex}(meV)	σ_c (meV)	σ_{ex}(meV)	A(OD)
1.642±0.001	11.0±0.05	23.9±0.05	31.1±0.07	0.590±0.001

Fitting parameters in Supplementary table 1. Considering the excitonic effect, the perovskite absorption spectrum near the bandedge can be described by Elliott's formula:[4-6]

$$A(\omega) = A_0 \cdot \theta(\hbar\omega - E_g) \cdot \left(\frac{\pi e^{\pi x}}{sinh(\pi x)}\right) + A_0 \cdot R_{ex} \sum_{n_{ex}=1}^{\infty} \frac{4\pi}{{n_{ex}}^3} \cdot \delta(\hbar\omega - E_g + R_{ex}/{n_{ex}}^2)$$

(Supplementary Equation 1)

where A_0 is a constant related to the transition matrix element, ω is the frequency of light, θ is the step function, E_g is the bandgap, x is defined as $R_{ex}^{1/2}/(\hbar\omega - E_g)^{1/2}$ where R_{ex} is the exciton binding energy, n_{ex} is the principal quantum number, and δ denotes a delta function. The first and second terms correspond to the continuum and excitonic absorption band, respectively. To account for inhomogeneous broadening, the continuum and excitonic part of Supplementary Equation 1 are convolved with Gaussian functions with standard deviation of σ_c and σ_{ex}, respectively. The full width at half maximum of the excitonic transition is then equal to $2.35\times\sigma_{ex}$.

Supplementary table 2. List of average pump intensities shown in the Fig.4 in main text. The pulse intensity is calculated as the ratio of average intensity to the pulse duration (~180 fs).

Average Intensity (μJ·cm^{-2})	4.6	6.5	8.3	11.2	15.9	21.9
Pulse Intensity (GW·cm^{-2})	0.026	0.036	0.046	0.062	0.088	0.12

Supplementary Note 1.

Quantum description of the optical Stark effect. Although no real excitons and/or free charge carriers will be generated with below-bandgap-pump due to the energy conservation, the exciton transition can be blue shifted *via* the optical Stark effect (OSE). Similar to the dressed-atom picture, the exciton OSE can be explained by the interaction between light and a two-level system. In the simplest case, the ground and exciton states are denoted as $|0\rangle$ and $|X\rangle$, respectively. The pump light with photon energy of $\hbar\omega_0$ is treated as a quantized electromagnetic field. The interaction between a quantized field of frequency v and a two level system is described by the Hamiltonian[7]:

$$H = H_0 + H^{(1)} = hva^{+}a + \frac{1}{2}\hbar\omega(|0\rangle\langle 0| - |X\rangle\langle X|) + \hbar g(|X\rangle\langle 0|a + a^{+}|0\rangle\langle X|)$$

(Supplementary Equation 2)

where H_0 represents the Hamiltonian of the unperturbed states and a radiation field mode, and $H^{(1)}$ accounts for the interaction part. In this expression, $\hbar\omega$ is the energy gap between $|X\rangle$ and $|0\rangle$. g is defined as $\frac{\mu_{0X}\mathcal{E}}{\hbar}$, where μ_{0X} is dipole transition matrix element, and ε has the dimensions of the electric field. The a^{+} and a are the photon creation and annihilation operators, respectively. When the photon energy is slightly detuned from the exciton transition energy (the detuning is denoted as Δ), the ground state dressed by n+1 (n>>1) photons, denoted as $|0, n+1\rangle$, is nearly degenerate with that of single exciton state with n photon, denoted as $|X, n\rangle$. These dressed states are the eigenstates of the unperturbed Hamiltonian. If we consider $H^{(1)}$ as a perturbation, the two states mentioned above will be driven apart. The energy shift of the exciton transition can be estimated as twice of the second-order correction of each state according to perturbation theory:

$$\delta E \approx 2 \cdot \frac{\left|\langle 0, n+1 \middle| H^{(1)} \middle| X, n\rangle\right|^2}{\Delta}$$

(Supplementary Equation 3)

Apply the perturbation Hamiltonian, we can get

$$\delta E \approx 2 \cdot \frac{(\hbar g)^2(n+1)}{\Delta} \approx \frac{(\mu_{0X})^2 2\varepsilon^2\left(n+\frac{1}{2}\right)}{\Delta} = \frac{(\mu_{0X})^2\langle F\rangle^2}{\Delta}$$

(Supplementary Equation 4)

where $\langle F\rangle$ is the average electric field of the radiation. It can then be easily see from Supplementary Equation (4) that the transition is blue (red) shifted for below (above) band gap excitation.

In addition to OSE, the energy shift in perovskites samples were also observed in electroabsorption measurement due to the static Stark effect.[8-11] However, the observed spectral change and energy shift are not consistent with the theory, which could be interpreted by our OSE measurement. For example, Supplementary

reference 11 shows that in the absence of strong permanent dipole the electroabsorption magnitude is quadratic to the applied field strength because the polarizability change dominates the energy shift. Under such condition, the electroabsorption spectrum should resemble the first derivative[12] rather than second derivative of the absorption spectrum (Figure 3 in Supplementary refence 11). On the other hand, when the electroabsorption magnitude of the perovskite samples is linear with the applied field,[9,10] it is indicative that a strong directional dipole exists between perovskite and charge transport contacts. In this case, the electroabsorption spectrum should also resemble the first derivative of the absorption spectrum, which was showed resembling the second derivative in Supplementary reference 9. The inconsistence between experimental observation and theoretical prediction is due to the unconsidered exciton transitions. Because near band gap the excition transition dominates the absorption spectrum,[2,5] the static Stark effect should mainly shift the exciton transitions. Considering the Gaussian-like line-shape of exciton transition, the elctroabsorption spectra measured in Supplementary reference 9 and 11 will resemble the first derivative of these exciton transitions, which is consistent with the static Stark effect description. In this paper, the OSE measurements in the perovskites will provide solid evidence that even in the absence of real excitons, the exciton transition can still play an important role in various spectroscopic characterizations.

Supplementary References:

1 Giovanni, D. *et al.* Highly Spin-Polarized Carrier Dynamics and Ultralarge Photoinduced Magnetization in $CH_3NH_3PbI_3$ Perovskite Thin Films. *Nano Lett.* **15**, 1553-1558 (2015).

2 Yang, Y. *et al.* Observation of a hot-phonon bottleneck in lead-iodide perovskites. *Nat Photon* **10**, 53-59 (2016).

3 Yang, Y. *et al.* Comparison of Recombination Dynamics in $CH_3NH_3PbBr_3$ and $CH_3NH_3PbI_3$ Perovskite Films: Influence of Exciton Binding Energy. *J Phys Chem Lett*, 4688-4692 (2015).

4 Elliott, R. J. Intensity of Optical Absorption by Excitons. *Phys. Rev.* **108**, 1384-1389 (1957).

5 Saba, M. *et al.* Correlated electron-hole plasma in organometal perovskites. *Nat Commun* **5**, 5049 (2014).

6 Yang, Y. *et al.* Low surface recombination velocity in solution-grown CH3NH3PbBr3 perovskite single crystal. *Nat Commun* **6**, 7961 (2015).

7 Scully, M. O. & Zubairy, M. S. *Quantum Optics*. (Cambridge University Press, 1997).

8 Deschler, F. *et al.* High Photoluminescence Efficiency and Optically Pumped Lasing in Solution-Processed Mixed Halide Perovskite Semiconductors. *J. Phys. Chem. Lett.* **5**, 1421-1426 (2014).

9 Li, C. *et al.* Iodine Migration and its Effect on Hysteresis in Perovskite Solar Cells. *Adv. Mater.* **28**, 2446-2454 (2016).

10 Roiati, V. *et al.* Stark effect in perovskite/TiO2 solar cells: evidence of local interfacial order. *Nano Lett.* **14**, 2168-2174 (2014).

11 Wu, X. *et al.* Composition-Dependent Light-Induced Dipole Moment Change in Organometal Halide Perovskites. *J. Phys. Chem. C* **119**, 1253-1259 (2015).

12 Sebastian, L., Weiser, G. & Bässler, H. Charge transfer transitions in solid tetracene and pentacene studied by electroabsorption. *Chem. Phys.* **61**, 125-135 (1981).
